# Successful resection of a subepithelial tumor at the duodenal bulbo-descending junction by endoscopic submucosal excavation

**DOI:** 10.1055/a-2749-3221

**Published:** 2025-12-11

**Authors:** Huanyu Wei, Mengyao Ji, Lei Yuan, Xu Huang, Lei Shen

**Affiliations:** 1 117921 Department of Gastroenterology, Renmin Hospital of Wuhan University, Wuhan, China; 2 Hubei Key Laboratory of Digestive Diseases, Wuhan, China; 3 117921 Department of Information Center, Wuhan University Renmin Hospital, Wuhan, China; 4 School of Automation, Nanjing University of Information Science and Technology, Nanjing, China

A 63-year-old woman was admitted to our hospital with “4 months of epigastric pain.” The epigastric pain was intermittent accompanied by belching and there was no vomiting, dizziness, palpitation or other discomfort. During conventional gastroscopy, a 2.0 cm × 1.8 cm mass was identified in the duodenal bulb ( Fig. 1 **a** ). Subsequent endoscopic ultrasonography revealed a well-circumscribed, hypoechoic, heterogeneous 1.15 cm × 1.29 cm mass arising from the fourth hyperechoic layer (muscularis propria) at the duodenal bulbo–descending junction, consistent with a preliminary diagnosis of gastrointestinal stromal tumor (GIST; Fig. 1 **b, c** ). Contrast-enhanced computed tomography of the whole abdomen showed a homogeneously hyper-enhancing 1.7 cm × 1.5 cm nodular lesion in the duodenal bulb, considered probably to be a subepithelial tumor ( Fig. 1 **d** ). Following multidisciplinary discussion and exclusion of contraindications, the patient underwent endoscopic submucosal excavation (ESE) of the lesion under general anaesthesia with endotracheal intubation.

**Fig. 1:**
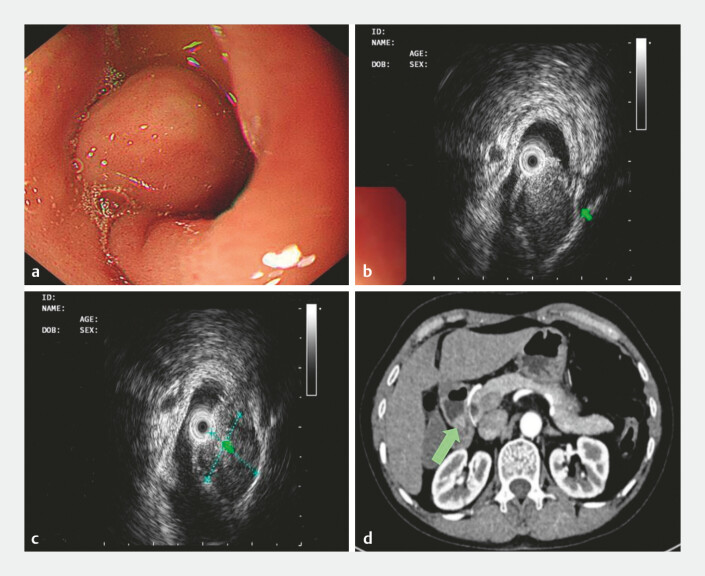
Preoperative endoscopic ultrasonography and contrast-enhanced CT examination results. **a** Using a gastroscope, a bulge was seen at the duodenal bulbo–descending junction. **b** The endoscopic ultrasonography showed that the lesion originated from the fourth low echo with uneven internal echo. **c** Endoscopy ultrasonography showed that the cross section of the lesion is 11.5 mm × 12.9 mm. **d** Transverse CT of the duodenal tumor. CT, computed tomography.

During the operation, a smooth surface bulge was seen at the duodenal bulbo–descending junction and the greater curvature. With the lesion clearly delineated in both the location and the extent, a mucosotomy was first created using a high-frequency electrosurgical knife (golden knife). Saline (0.9%) was then infused continuously through the incision while the dissection was advanced, generating a submucosal fluid cushion that facilitated atraumatic separation of the mucosal layer from the underlying submucosa. The tumor exhibited an exophytic growth pattern. By gently displacing the tumor with the transparent cap mounted on the tip of the endoscope, the basal aspect of the lesion was fully exposed. Subsequently, the tumor was transected at its base with a mucosal incision knife (woodpecker knife) in conjunction with a snare, enabling en-bloc resection of the entire tumor ( Fig. 2 **a–c** ).

**Fig. 2:**
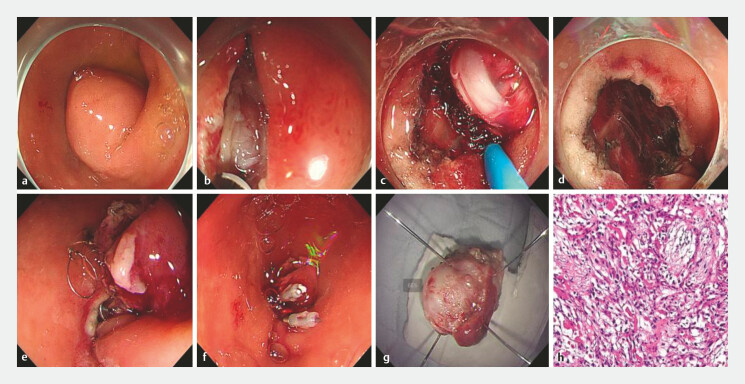
Endoscopic submucosal dissection of a tumor at the duodenal bulbo–descending junction. **a** The lesion of the duodenum. **b** Incise the mucosa to expose the tumor with a golden knife. **c** Complete en bloc resection is achieved with a woodpecker knife. **d** The resection bed is meticulously coagulated. **e** The mucosal defect was closed with one OTS clip. **f** Two long titanium clips and one hemostatic clip were added to successfully close the defect. **g** The tumor measured 1.9 × 1.8 × 1.6 cm in size. **h** Pathology revealed a gastrointestinal stromal tumor.

After complete endoscopic resection, the resection bed showed active bleeding. The transparent cap attached to the tip of the endoscope was used to compress the bowel wall, allowing preliminary mapping of the bleeding site. The target site was then clamped with a hemostatic forceps, and immediate cessation of bleeding confirmed the exact bleeding point. The mucosa was next lifted toward the lumen and meticulously coagulated with the hemostatic forceps. Because the duodenal wall is extremely thin, soft-coagulation was performed with low power and brief, intermittent applications to avoid transmural injury and intra-operative perforation. After coagulating the bleeding arteriole, focal oozing persisted, and we used the hemostatic forceps for refined pinpoint coagulation until complete hemostasis of the resection bed was secured ( Fig. 2 **d** ).

Finally, the mucosal defect was closed with one over-the-scope clip (OTS clip), two long titanium clips, and one hemostatic clip, and a nasogastric tube was left in situ for postoperative decompression ( Fig. 2 **e, f** ). The specimen, measuring 1.9 cm × 1.8 cm × 1.6 cm, was retrieved en-bloc and sent for full-thickness histopathologic examination ( Video 1 ). Pathology revealed a GIST, confirming the diagnosis of benign duodenal neoplasm ( Fig. 2 **g, h** ).

Successful resection of a subepithelial tumor at the duodenal bulbo–descending junction by endoscopic submucosal excavation.Video 1

On postoperative day 3, the nasogastric tube was removed and the patient was advanced to a liquid diet without any evidence of delayed bleeding or other complications, discharge on postoperative day 5. After 2 months of convalescence, the patient returned for scheduled clip removal to prevent hyperplasia of the surgical site and resulting stenosis of the lumen. Endoscopic retrieval of the OTS clip was performed without difficulty, and thorough irrigation revealed no active bleeding, confirming complete mucosal healing ( Fig. 3 ).

**Fig. 3:**
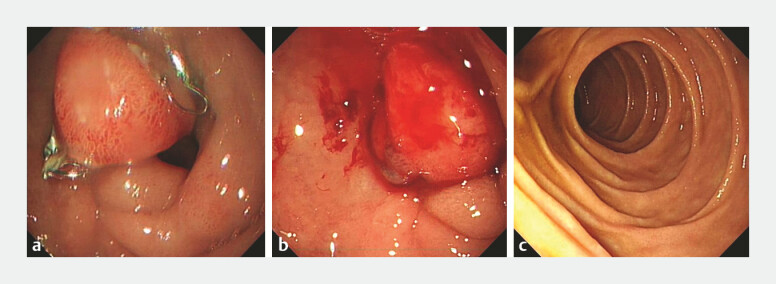
An OTS clip was removed. **a** An OTS clip is in place. **b** Minor bleeding in the duodenal bulb after the clip is removed. **c** After removing the OTS clip, the endoscope can pass through the duodenum smoothly.

Primary duodenal GISTs account for only 3–5% of all gastrointestinal stromal tumors, and those arising at the bulb–descending junction are even rarer. The unique anatomy of the duodenal bulbo–descending junction, narrow lumen, thin wall, limited endoscopic working space, rich vasculature, and proximity to the ampulla and pancreatic parenchyma, renders ESE exceptionally challenging 1 2 . At the same time, we selected the use of OTS clip clamping incision, which can prevent ischemic necrosis of deep structures, and has a good application scenario for endoscopic surgery to remove deep tumors. The OTS clip is a novel titanium-nickel alloy device designed for endoscopic defect closure and hemostasis in the gastrointestinal tract. Compared with conventional through-the-scope clips, the OTS clip system delivers markedly higher grasping and compressive forces, enabling rapid, effective, and durable hemostasis 3 4 . In addition, the clip’s tooth-like design preserves subjacent blood and lymphatic flow, thereby minimizing the risk of necrosis at the lesion base 5 .

We report the complete ESE en bloc resection of a duodenal bulbo–descending junction GIST, demonstrating technical feasibility when expertise and caution are maximal.

Endoscopy_UCTN_Code_TTT_1AO_2AG_3AD

## Notes

Endoscopy E-Videos https://eref.thieme.de/e-videos
*E-Videos* is an open access online section of the journal *Endoscopy* , reporting on interesting cases and new techniques in gastroenterological endoscopy. All papers include a high-quality video and are published with a Creative Commons CC-BY license. Endoscopy E-Videos qualify for HINARI discounts and waivers and eligibility is automatically checked during the submission process. We grant 100% waivers to articles whose corresponding authors are based in Group A countries and 50% waivers to those who are based in Group B countries as classified by Research4Life (see: https://www.research4life.org/access/eligibility/ ). This section has its own submission website at https://mc.manuscriptcentral.com/e-videos .
